# Modulation of Cyclins, p53 and Mitogen-Activated Protein Kinases Signaling in Breast Cancer Cell Lines by 4-(3,4,5-Trimethoxyphenoxy)benzoic Acid

**DOI:** 10.3390/ijms15010743

**Published:** 2014-01-08

**Authors:** Kuan-Han Lee, Wen-Yueh Ho, Shu-Jing Wu, Hany A. Omar, Po-Jui Huang, Clay C. C. Wang, Jui-Hsiang Hung

**Affiliations:** 1 Department of Pharmacy, Chia Nan University of Pharmacy and Science, Tainan 71710, Taiwan; E-Mail: kuanhanlee@mail.chna.edu.tw; 2 Department of Cosmetic Science and Institute of Cosmetic Science, Chia Nan University of Pharmacy and Science, Tainan 71710, Taiwan; E-Mail: rickho@mail.chna.edu.tw; 3 Department of Nutritional Health, Chia Nan University of Pharmacy and Science, Tainan 71710, Taiwan; E-Mail: wsj268@mail.chna.edu.tw; 4 Department of Pharmacology, Faculty of Pharmacy, Beni-Suef University, Beni-Suef 62514, Egypt; E-Mail: omar.22@buckeyemail.osu.edu; 5 Department of Pharmacology, College of Pharmacy, University of Sharjah, Sharjah 27272, UAE; 6 Department of Medicinal and Applied Chemistry, College of Life Science, Kaohsiung Medical University, Kaohsiung 807, Taiwan; E-Mail: brhuang@kmu.edu.tw; 7 Department of Pharmacology and Pharmaceutical Sciences, University of Southern California, School of Pharmacy, Los Angeles, CA 90089, USA; E-Mail: clayw@usc.edu; 8 Department of Biotechnology, Chia Nan University of Pharmacy and Science, Tainan 71710, Taiwan; 9 Drug Discovery and Development Center, Chia Nan University of Pharmacy and Science, Tainan 71710, Taiwan

**Keywords:** 4-(3,4,5-trimethoxyphenoxy)benzoic acid, MCF-7, MDA-468, apoptosis, MAPK kinases, p53, cyclins

## Abstract

Despite the advances in cancer therapy and early detection, breast cancer remains a leading cause of cancer-related deaths among females worldwide. The aim of the current study was to investigate the antitumor activity of a novel compound, 4-(3,4,5-trimethoxyphenoxy)benzoic acid (TMPBA) and its mechanism of action, in breast cancer. Results indicated the relatively high sensitivity of human breast cancer cell-7 and MDA-468 cells towards TMPBA with *IC*_50_ values of 5.9 and 7.9 μM, respectively compared to hepatocarcinoma cell line Huh-7, hepatocarcinoma cell line HepG2, and cervical cancer cell line Hela cells. Mechanistically, TMPBA induced apoptotic cell death in MCF-7 cells as indicated by 4′,6-diamidino-2-phenylindole (DAPI) nuclear staining, cell cycle analysis and the activation of caspase-3. Western blot analysis revealed the ability of TMPBA to target pathways mediated by mitogen-activated protein (MAP) kinases, 5′ adenosine monophosphate-activated protein kinase (AMPK), and p53, of which the concerted action underlined its antitumor efficacy. In addition, TMPBA induced alteration of cyclin proteins’ expression and consequently modulated the cell cycle. Taken together, the current study underscores evidence that TMPBA induces apoptosis in breast cancer cells via the modulation of cyclins and p53 expression as well as the modulation of AMPK and mitogen-activated protein kinases (MAPK) signaling. These findings support TMPBA’s clinical promise as a potential candidate for breast cancer therapy.

## 1. Introduction

Breast cancer is one of the most common and deleterious of all diseases affecting females in addition to being the second leading cause of death among women worldwide [1,2]. Many factors are associated with the increased risk of breast cancer such as gene mutation, radiation, lifestyle and alcohol intake [3–7]. While many breast cancer patients initially respond to chemotherapy, resistance often rapidly develops which leads to poor clinical prognosis [8]. Breast cancer resists most of the clinically-available anticancer agents during the course of therapy which underscores the current need for novel, easily accessible drugs with superior efficacy.

Apoptosis is a highly regulated process of programmed cell death, and disruption of this process represents a major contributing factor in the pathology of cancer [9]. Apoptosis is mediated by the action of caspases, a group of cysteine proteases which can be activated through two pathways, extrinsic and intrinsic pathways [10–13]. The activation of caspases induces protein cleavage which results in chromatin condensation, DNA fragmentation, and cell shrinkage [14]. Another major player in apoptosis process is p53 which is activated when mammalian cells are subjected to stress conditions such as hypoxia, radiation, DNA damage or chemotherapeutic drugs [15,16]. The main function of the tumor suppressor, p53, is to limit the cellular proliferation by inducing cell cycle arrest and apoptosis in response to stress. In addition to its role in suppressing tumorigenesis, p53 contributes to chemotherapy-induced cell death [17]. p53 mediates apoptosis through a linear pathway involving Bax transactivation, mitochondrial cytochrome c release mitochondria and caspase-9 activation, followed by the activation of caspase-3, -6, and -7 [18,19].

Pharmacological induction of cell cycle arrest especially in transformed cells is an effective strategy in restricting tumor growth both *in vitro* and *in vivo* [20,21]. The cell cycle is regulated by cyclic formation and breakdown of various cyclin-cyclin-dependent kinase (CDK) complexes [22,23]. Cyclins are key regulators of the mammalian cell cycle, functioning primarily in concert with their catalytic partners, the cyclin-dependent kinases (CDKs). Overexpression of cyclin proteins has been linked to human cancer [22,23]. Therefore, the ability of these cyclins to activate CDKs is the most extensively documented mechanism for their oncogenic actions and provides an attractive therapeutic target [24–26].

4-(3,4,5-Trimethoxyphenoxy)benzoic acid was synthesized and the mechanisms of TMPBA-induced cell death are still unclear. Therefore, the aim of the current work was to extensively explore the cytotoxic activity of TMPBA against human breast cancer cells and to investigate the underlying mechanism of action. Our results demonstrated that TMPBA induced apoptosis by targeting a broad range of signaling pathways, including those mediated by MAP kinases, p53, cell cycle regulatory proteins and AMPK. To the best of our knowledge, this is the first study investigating the anticancer activity and underlying mechanisms of TMPBA in breast cancer cell lines.

## 2. Results and Discussion

### 2.1. Differential Susceptibility of Cancer Cell Lines to TMPBA-Induced Cell Death

**Figure 1.**
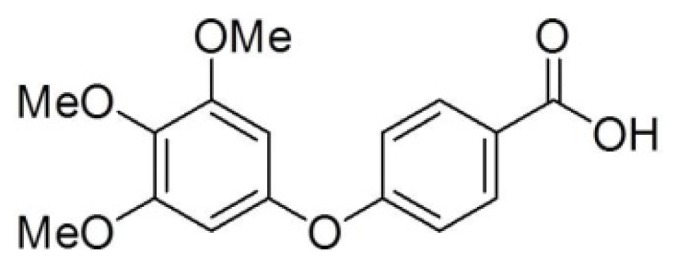
The structure of 4-(3,4,5-trimethoxyphenoxy)benzoic acid (TMPBA).

To investigate the anti-proliferative effects of TMPBA (Figure 1), five human cancer cell lines were examined in response to TMPBA treatment: Huh-7, HepG2, Hela, MCF-7, and MDA-468.

**Figure 2.**
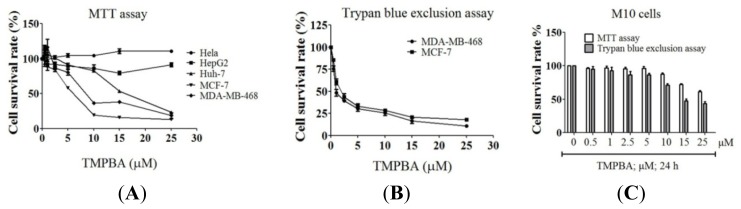
The anti-proliferative activity of TMPBA in Hela, HepG2, Huh-7, MCF-7 and MDA468 cells. (**A**) The effect of TMPBA on the cell viability of different cancer cell lines was assessed by MTT assay after treatment for 24 h. Points, mean; bars, SD (*n* = 6); (**B**) The effects of TMPBA on MCF-7 and MDA-MB-468 cells were assessed by trypan blue exclusion assay after treatment for 24 h. Points, mean; bars, SD (*n* = 6); (**C**) The effects of TMPBA on the cell viability of M10 cell line was assessed by MTT assay and trypan blue exclusion assay after treatment for 24 h. Points, mean; bars, SD (*n* = 6).

The cell lines were treated with TMPBA at the indicated doses for 24 h and the cell viability was determined by MTT assay. As shown in Figure 2, results indicated that HepG2, Huh-7, and Hela cancer cell lines were more resistant to TMPBA. On the other hand, the breast cancer cell lines MCF-7 and MDA-468 were very sensitive to TMPBA. The IC_50_ values of TMPBA for MCF-7 and MDA-468 cells were 5.9 and 7.9 μmol/L, respectively. Furthermore, TMPBA-induced MCF-7 and MDA-MB-468 cell death were confirmed by trypan blue exclusion assay (Figure 2B). In contrast, human normal mammary epithelial M10 cells were not susceptible to the cytotoxic effect of TMPBA (Figure 2C).

### 2.2. TMPBA Changed Cell Morphology and Decreased Colony Formation in Breast Cancer Cells

**Figure 3.**
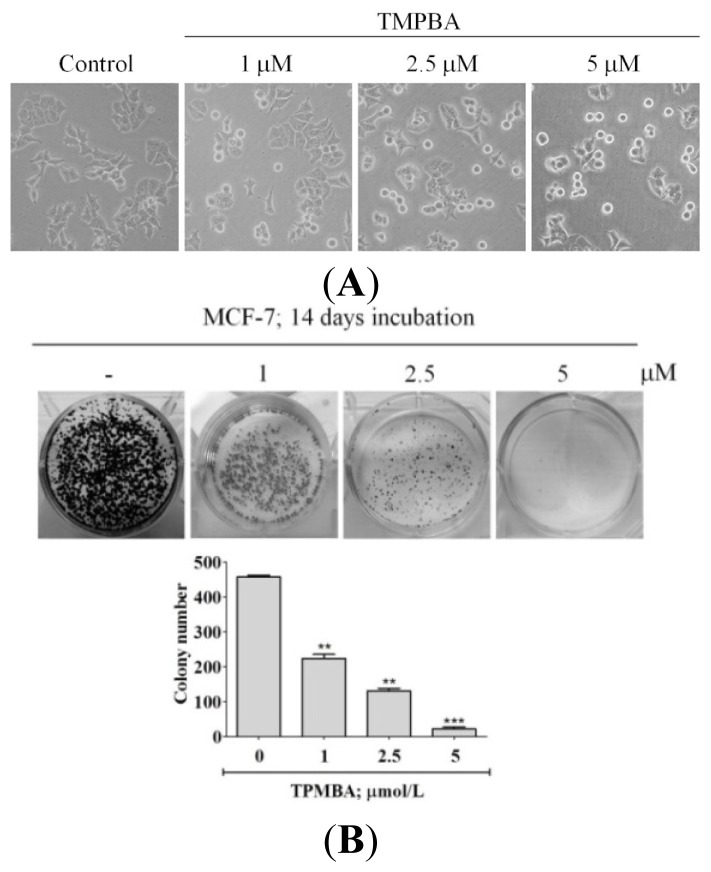
TMPBA changed cell morphology and decreased colony formation ability of MCF-7 cells. (**A**) The morphological changes after a 24-hour TMPBA treatment with MCF-7 cells. The cells were followed by photography under phase-contrast magnification (200×); (**B**) MCF-7 cells were treated with compounds, and colony formation was scored after 14 days. The number of colonies in the graphs was representative of three independent experiments (lower panel). Data represent the mean ± SD (*n* = 3). Significant differences (**, *p* < 0.01, ***, *p* < 0.001) between the control and experimental group are marked with asterisks.

To confirm the ability of TMPBA to induce cell death in breast cancer cells, MCF-7 cells were treated with TMPBA then were observed for changes in the cell morphology and colony formation ability. For colony-forming ability of MCF-7 cells, cells were exposed to TMPBA then after 14 days; grown colonies were stained with crystal violet and counted. Results indicated that TMPBA caused progressive morphological changes from flat to round (Figure 3A). In addition, TMPBA significantly decreased colony formation ability of MCF-7 cells (Figure 3B). These results confirm the ability of TMPBA to target breast cancer cells.

### 2.3. Induction of Cell Apoptosis and G2/M Cell Cycle Arrest by TMPBA Treatment in Breast Cancer Cells

**Figure 4.**
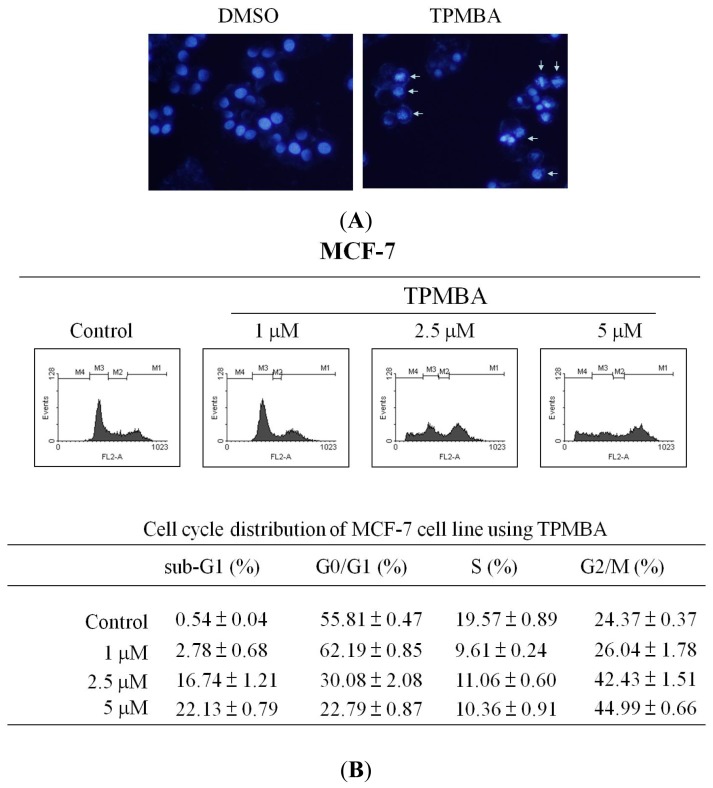

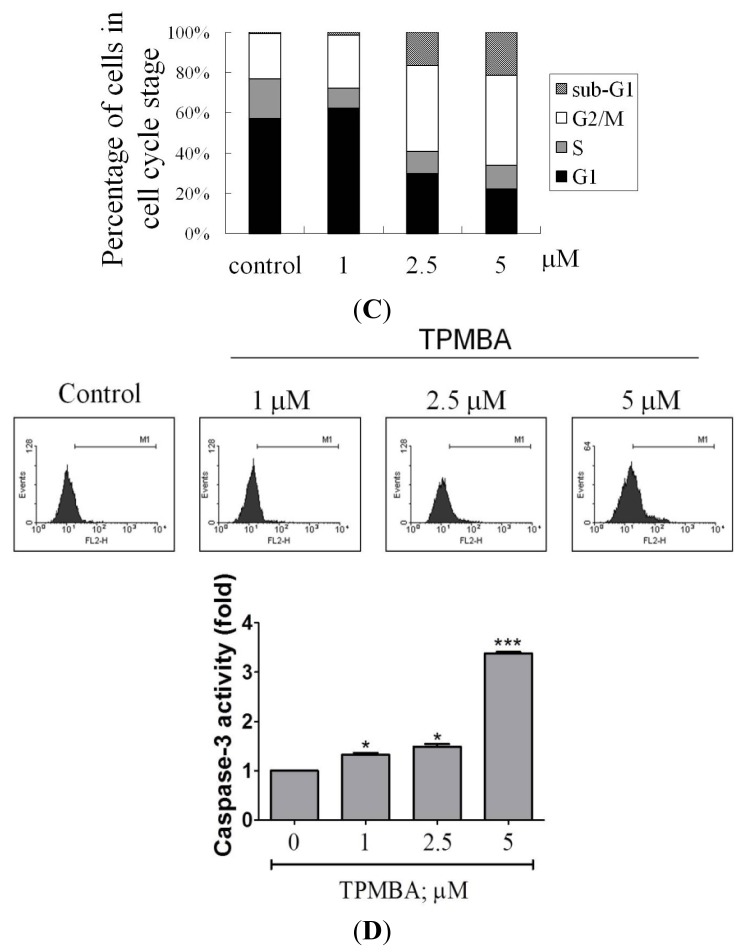
TMPBA induced G2/M arrest and apoptosis in MCF-7 cells. (**A**) MCF-7 cells were treated with 5 μM TMPBA for 12 h, the morphological changes were being analyzed by fluorescence microscopy with DAPI staining. Cells had nuclei maintaining mesh-like structure of chromatin or condensed chromatin (arrow); (**B**) Flow cytometric analysis of cell cycle in MCF-7 cells with TMPBA in 10% FBS-containing DMEM for 12 h. MCF-7 cells were cultured with TMPBA for the dose indicated. The cells were analyzed by flow cytometry after staining with propidium iodide (PI); (**C**) The percentages in the graphs represent the percent of cell cycle phases in the respective quadrants (M1:G2/M phase; M2: S phase; M3: G1 phase; M4: Sub-G1 phase). Columns, mean; bars, SD; (**D**) Flow cytometric analysis of dose-dependent effect of TMPBA on caspase-3 activity in MCF-7 cells. The relative caspase-3 activities, normalized to DMSO control, at the indicated concentrations of the compound. *Columns*, mean of three independent experiments; bars, SD (*n* = 3). Significant differences (* *p* < 0.05, *** *p* < 0.001) between the control and experimental group are marked with asterisks.

To investigate the mechanism of TMPBA-induced cell death in breast cancer cells, the ability of TMPBA to induce apoptosis was tested initially by DAPI staining. As shown in Figure 4A, chromatin condensation and apoptotic bodies were clearly observed in TMPBA-treated cells. Second, the effects of TMPBA on cell cycle progression in breast cancer cells was tested using DNA flow cytometric analysis. Results revealed that TMPBA caused the accumulation of MCF-7 cells in G2/M and sub-G1 phase cells in a dose-dependent manner (Figure 4B). It was observed that as the percentage of cells in G2/M increased, the percentage of cells in G1 phase decreased, and the proportion of S-phase cells was not significantly altered by TMPBA treatment in MCF-7 cells (Figure 4C). Third, since caspases play a pivotal role in apoptosis, the ability of TMPBA to activate caspases-3 was investigated using flow cytometric analysis. As shown in Figure 4D, the exposure of MCF-7 cells to TMPBA caused a dose-dependent activation of caspase-3 activity which reached a 3.3-fold increase at 5 μM compared to control cells. Collectively, these results highlighted the mechanism of TMPBA-induced cell death in breast cancer cells to be, at least in part, via the induction of apoptosis.

### 2.4. Modulation of the Expression of Cyclin B, Cyclin E, cdc2, and cdc25 in TMPBA-Treated Cells

**Figure 5.**
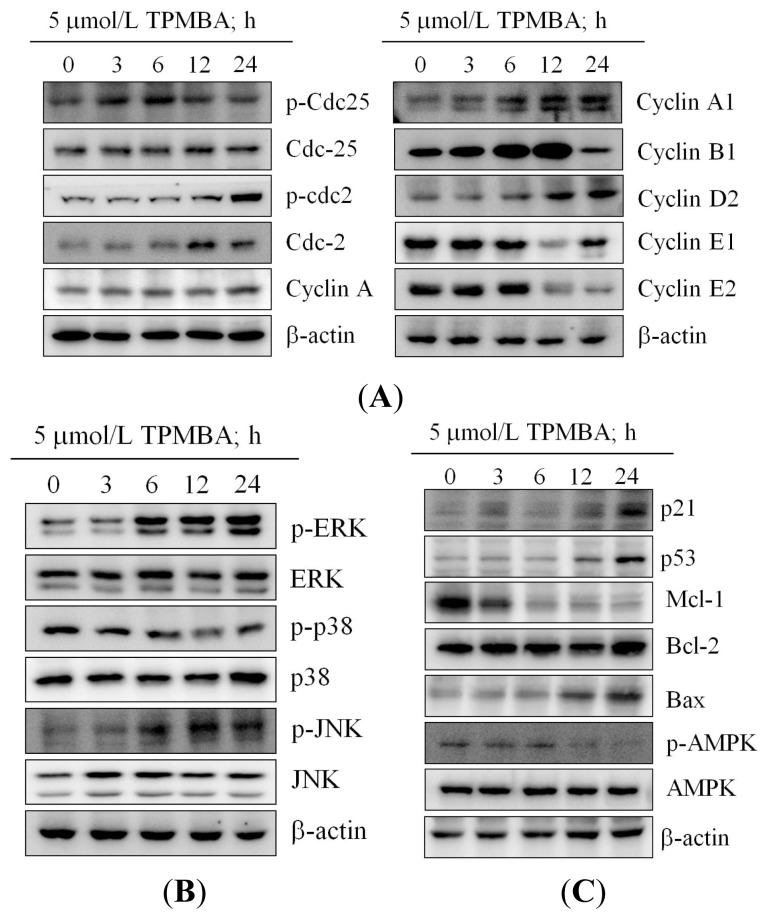
Western blot analysis of the time-dependent modification effects of TMPBA on the expression and/or phosphorylation. MCF-7 Cells were exposed to 5 μM TMPBA in 10% FBS-supplemented DMEM for the indicated time intervals. (**A**) The effect of TMPBA on MAP kinases ERK1/2, JNK and p38 activation was investigated in MCF-7 cells; (**B**) Regulation of cell cycle-regulatory proteins by TMPBA, including cdc2, cdc25, cyclin A, cyclin A1, cyclin B1, cyclin D2, cyclin E1, cyclin E2, was determined by Western blotting; (**C**) Apoptosis-associated proteins p21, p53, MCl-1 Bcl-2, Bax, and AMPK were evaluated in TMPBA-induced cell apoptosis.

To investigate the mechanism of TMPBA-induced modulation of cell cycle progression, Western blot analysis was used to test the effect of TMPBA on cyclin proteins expression. Results indicated the ability of TMPBA to down-regulate cyclin A, cyclin A1, cyclin B1, cyclin E1 and cyclin E2 expression (Figure 5A). On the other hand, TMPBA treatment increased the phosphorylated cdc2 (Tyr 15) and cdc25 (Ser 216) in MCF-7 cells (Figure 5A). These results could explain the accumulation of a high percentage of cells at G2/M and cell cycle arrest that were observed by flow cytometry.

### 2.5. Effect of TMPBA on MAPK Signaling Pathways

Since mitogen-activated protein kinase (MAPK) families have been reported to play an important role in cell cycle regulation [27], and the effect of TMPBA on MAPK kinases signaling pathways was evaluated. As shown in Figure 5B, TMPBA treatment significantly induced the phosphorylation of ERK and JNK while inhibited the phosphorylation of p38 in MCF-7 cells in a time-dependent manner. These results confirm the involvement of MAPK signaling pathways in the TMPBA-induced cell cycle modulation in breast cancer.

### 2.6. TMPBA Effect on the Expression of p21, p53, Bax, Bcl-2, MCL-1 and AMPK

To investigate the mechanism of TMPBA-induced apoptosis, the effect of TMPBA on p53, p21, Bcl-2, Mcl-1 and Bax levels were tested. As shown in Figure 5C, Western blot analysis revealed that TMPBA treatment caused a decrease in Mcl-1 levels and an increase in Bax, p21 and p53 levels compared to control cells. On the other hand, the expression of Bcl-2 was not affected by TMPBA treatment. In addition, TMPBA decreased the phosphorylation of AMPK (Figure 5C). These results partially explained the mechanism of TMPBA-induced G2/M cell cycle arrest and the consequent apoptosis.

### 2.7. TMPBA-Induced p53 Expression via NF-κB Signaling Pathway

**Figure 6.**
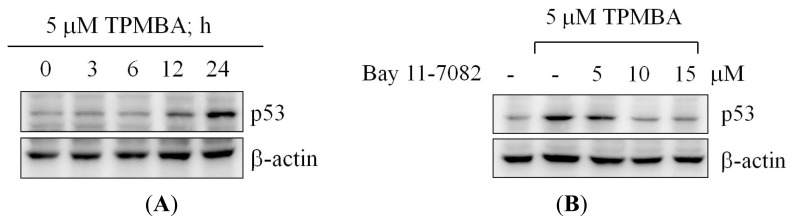
Regulation of p53 expression depends on NF-κB signaling pathway. (**A**) The time-dependent effect of TMPBA on p53 expression. MCF-7 Cells were exposed to TMPBA intoTMPBAin 10%FBS-supplemented DMEM for the indicated time intervals. The cell lysates were analyzed by western blotting with antibodies for p53 and β-actin. The p53 protein expression level was quantified densitometrically; (**B**) The effect of the pharmacological inhibitor of NF-κB, Bay 11-7082, on TMPBA-induced p53 expression. Cultures of MCF-7 cells were treated with 5 μM TMPBA in the presence of Bay 11-7082. The p53 protein expression level was quantified densitometrically.

Since TMPBA treatment significantly increased p53 expression (Figure 6A) and based on the role of NF-κB family members in the regulation of p53 gene in response to stress [28,29]; the role of NF-κB signaling pathway in p53 modulation was investigated. We hypothesized that activation of NF-κB may be the mechanism that accounts for the increased expression of p53 during TMPBA treatment. Results indicated that co-treatment of NF-κB inhibitor, Bay11-7028, with TMPBA, dramatically inhibited TMPBA-induced expression of p53 (Figure 6B). These results confirmed the role of NF-κB signaling pathway in TMPBA-induced p53 expression.

## 3. Discussion

**Figure 7.**
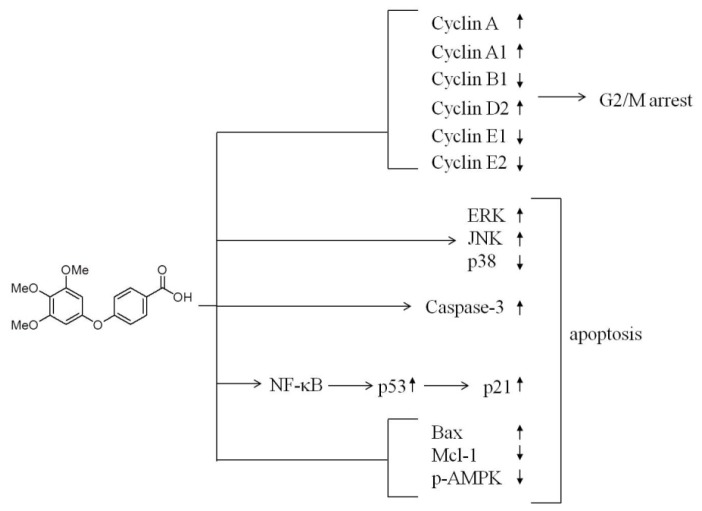
Diagram depicting the effect of TMPBA on cell cycle regulatory proteins, MAP kinases, and p53. The interplay between these signaling networks at different cellular levels results in the ability of TMPBA to induce apoptosis in MCF-7 breast cancer cells.

Here, we report the translational potential of TMPBA to be developed into a new therapeutic agent for breast cancer. TMPBA effectively induced apoptotic cell death in MCF-7 and MDA-468 breast cancer cell lines via the modulation of multiple signaling pathways. The unique pleiotropic mode of action of TMPBA by targeting signaling pathways that regulate cancer cell survival and progression such as cell cycle-regulatory proteins, AMPK, MAPK kinase, caspase-3 activity, p53 and Bcl-2 family proteins led to G2/M cell cycle arrest and apoptosis (Figure 7). In addition, the induction of p53 expression in response to TMPBA treatment via NF-κB signaling pathway underscored its clinical potential as a chemopreventive agent for breast cancer [30].

During the process of carcinogenesis and tumor progression, tumor cells upregulate multiple signaling pathways controlling cell proliferation, survival, invasion, and metastasis [29,31]. The modulation of these signaling pathways usually affects cellular sensitivity to chemotherapeutic drugs and induces resistance and, thus, affects the outcome of cancer treatment [32]. The simultaneous targeting of multiple cancer cell survival pathways is one of the most successful strategies to minimize resistance in cancer therapy [33,34]. The unique pleiotropic mechanism of action of TMPBA offers a valuable outcome via simultaneous targeting of cancer cell growth and inhibiting resistance.

Since p53 tumor suppressor functions as a regulator of transcription and mediates several biological effects, such as growth arrest and apoptosis in response to various forms of stress [35–37], we investigated the effect of TMPBA on p53 expression. Results showed the ability of TMPBA to significantly induce the expression p53 through NF-κB signaling pathway. The activation of NF-κB and the induction of p53 expression are essential therapeutic effects of many clinical cancer drugs [38–40].

The inhibition of AMPK phosphorylation by TMPBA treatment in MCF-7 cells highlighted the involvement of AMPK in TMPBA-induced cell death. AMPK emerged as a key kinase controlling many cellular processes, particularly pathways involved in cellular energy status, growth and/or survival of cancer cells [41–44]. In addition, the pro-apoptotic effect of AMPK has been attributed to the inhibition of cell cycle progression, activation of the c-Jun NH(2)-terminal kinase (JNK) pathway, caspase-3 activation, and up-regulation of the pro-apoptotic p53 protein [42]. These findings prompted us to confirm the pro-apoptotic activity of AMPK in response to TMPBA treatment by studying key players involved in cell cycle regulation and apoptosis such as cyclin proteins expression and MAPK signaling. Our cell cycle and western blot analyses revealed the ability of TMPBA to cause cell cycle arrest and MAPK signaling modulation leading to cancer cell apoptosis.

In summary, the current study provided an insight into the mechanism of breast cancer chemotherapy activity of TMPBA. Results indicated that TMPBA is a pleiotropic agent that induced cancer cell death by modulating multiple signaling pathways involved in cell cycle regulation, stress response and apoptosis. These findings support the clinical development of TMPBA into a new therapeutic component for breast cancer.

## 4. Experimental Section

### 4.1. Cell Culture and Material

Huh-7, HepG2, Hela, MCF-7, and MDA-468 cell lines were obtained from the American Type Culture Collection (ATCC). M10 human normal mammary epithelial cell line was the gift from Shyng-Shiou Yuan. Cells were maintained at 37 °C in a 5% CO_2_ atmosphere in DMEM supplemented with 10% heat-inactivated fetal bovine serum, 2 mM l-glutamine, 100 units/mL penicillin, and 100 μg/mL streptomycin. 2,5-diphenyl-tetrazolium bromide (MTT) and propidium iodide (PI) were obtained from Sigma-Aldrich (St. Louis, MO, USA). Antibodies against various proteins were obtained from the following sources: cdc25, p-cdc25, cdc2, p-cdc2, p53, p21, Bcl-2, Mcl-1, Bax, and β-actin (Santa Cruz Biotechnology, Dallas, TX, USA); cyclin A, cyclin A1, cyclin A2, cyclin B1, cyclin D1, cyclin D2, cyclin D3, cyclin E1, cyclin E2, cyclin H, ERK, p-ERK, p38, p-p38, JNK, p-JNK, AMPK, p-AMPK, anti-rabbit IgG-horseradish peroxidase (HRP) conjugates, rabbit anti-mouse IgG-HRP conjugates antibodies (Cell Signaling, Danvers, MA, USA).

### 4.2. Cell Viability

Cell viability was assessed using the MTT assay in three replicates as mentioned before [33]. Briefly, Huh-7, HepG2, Hela, MCF-7, and MDA-468 cells were seeded at 5 × 10^3^ per well in 96-well flat-bottomed plates and incubated in 10% FBS-supplemented DMEM for 16 h. Cells were treated with TMPBA at the indicated doses while controls received vehicle (DMSO). After 72 h, the drug-containing medium was replaced with 20 μL of 10% FBS-supplemented DMEM containing 0.5 mg/mL MTT, and cells were incubated in the CO_2_ incubator at 37 °C for 4 h. Medium was removed, the reduced MTT was solubilized in 100 μL of DMSO per well, and each well was transferred to 96-well plates to measure absorbance at 570 nm.

### 4.3. Trypan Blue Exclusion Method

Cell survival was determined by trypan blue exclusion method. Briefly, MCF-7 and MDA-MB-468 cells were seeded at 2 × 10^4^ per well in 24-well flat-bottomed plates and incubated in 10% FBS-supplemented DMEM for 16 h. Cells were treated with TMPBA at the indicated concentrations while controls received vehicle (DMSO). Cells were treated for 24 h and then collected by trypsinization. Viable cells were counted by an automated cell counter. Cells restricting trypan blue entry were considered viable.

### 4.4. Colony Formation Assay

For the colony formation assay, MCF-7 cells were seeded at 3 × 10^3^ per well in six-well, flat-bottomed plates and incubated in 10% FBS-supplemented DMEM for 16 h. The cells were then treated with different concentrations of TMPBA for 24 h. The culture medium was replenished, and cells were maintained at 37 °C for 10 days with medium changed every other day. Grown colonies were fixed with 3.7% formaldehyde and stained with crystal violet. The number of cell colonies was determined directly on each well.

### 4.5. Morphological Observation of Nuclear Changes

The morphological changes of nuclear chromatin in cells undergoing apoptosis were detected by staining with DAPI as mentioned before [45]. Briefly, 5 × 10^5^ cells/dish were plated onto 6-cm dishes and incubated at 37 °C for 16 h. Cells were then treated with 5 mM TMPBA for 12 h. After treatment, the cells were fixed with 3% paraformaldehyde for 15 min, and staining with DAPI for 2 min. Apoptotic nuclei were identified by reduced nuclear size, condensed chromatin gathered at the periphery of the nuclear membrane or by a total fragmented morphology of nuclear bodies.

### 4.6. Cell Cycle Analysis

To determine cell-cycle distribution, 5 × 10^5^ cells in a 6-cm dish were treated with various concentrations of TMPBA for 24 h. After incubation, the supernatant was removed, and the cells were then fixed in 70% ethanol/PBS, pelleted, and re-suspended in buffer containing RNase A and propidium iodide. Cell-cycle distribution was determined by flow cytometry analysis, and the percentages of cells were determined using the WinMDI software (version 2.8, Joe Trotter, Scripps Research Insititute, La Jolla, CA, USA, 2000).

### 4.7. Analysis of Caspase-3 Activity

Caspase-3 activity was determined using PE active caspase-3 apoptosis kit (BD Pharmingen, San Jose, CA, USA). Briefly, MCF-7 (5 × 10^5^) cells in 10-cm dishes were subjected to drug treatment as indicated for 72 h and were re-suspended in 0.5 mL Cytofix/Cytoperm solution for 20 min on ice. Cells were then incubated in 100 μL of Perm/Wash buffer containing 20 μL caspase-3 antibodies for 30 min at room temperature. Each sample was then added to 400 μL Perm/Wash buffer, and caspase-3 activity signals were analyzed by flow cytometry (BD Biosciences, San Jose, CA, USA)

### 4.8. Western Blot Analysis

The cell lysates were collected with RIPA lysis buffer (50 mM Tris-Cl pH 7.4, 150 mM NaCl, 1% NP40, 0.25% Na-deoxycholate, 1 mM PMSF, 1 mM EDTA, 5 μg/mL Aprotinin) containing protease inhibitors (1 mM PMSF, 1 mM orthovanadate, 1 mM EDTA, and 10 μg/mL leupeptin). Protein concentrations of cell lysates were measured using a Micro BCA protein assay reagent kit (Pierce, Rockford, IL, USA). To the cell lysate, the same volume of SDS-PAGE loading buffer (100 mmol/L Tris-HCl, 4% SDS, 5% β-mercaptoethanol, 20% glycerol, and 0.1% bromphenol blue (pH 6.8)) was added, and the cell lysates were boiled for 10 min. Equal amounts of proteins were resolved in SDS-polyacrylamide gels and transferred to nitrocellulose membranes using a semi-dry transfer cell. The blotted membranes were washed twice with TBS containing 0.1% Tween 20 (TBST; 10 mM Tris-HCl, pH 7.5, 150 mM NaCl, 0.05% Tween-20). After blocking with TBST containing 5% nonfat milk for 1 h, the membranes were probed with antibodies against: cdc25, p-cdc25, cdc2, p-cdc2, p53, p21, Bcl-2, Mcl-1, Bax, cyclin A, cyclin A1, cyclin A2, cyclin B1, cyclin D1, cyclin D2, cyclin D3, cyclin E1, cyclin E2, cyclin H, ERK, p-ERK, p38, p-p38, JNK, p-JNK, AMPK, p-AMPK, and β-actin antibodies in 1% TBST nonfat milk at 4 °C overnight. The membranes were then washed thrice with TBST for a total of 15 min. The secondary anti-mouse IgG-HRP conjugates or anti-rabbit IgG-HRP conjugates (1:2000 dilutions) was subsequently incubated with the membranes for 1 h at room temperature and the membranes were then washed extensively for 50 min with TBST. The blots were visualized with the enhanced chemiluminescence (GE, Pittsburgh, PA, USA) according to the manufacturer’s instructions.

### 4.9. Statistical Analysis

Results were presented as mean ± SD of three independent experiments in triplicates and analyzed by Student’s *t* test. Differences were considered significant at * *p* < 0.05 and ** *p* < 0.01, respectively.

## 5. Conclusions

In the present study, we established TMPBA as an anti-breast cancer agent. Our results have improved our understanding of the potential effects of TMPBA on breast cancer cells. We believe that our findings provide a foundation for further studies on TMPBA as a new therapeutic candidate for cancer.
